# A Python Clustering Analysis Protocol of Genes Expression Data Sets

**DOI:** 10.3390/genes13101839

**Published:** 2022-10-12

**Authors:** Giuseppe Agapito, Marianna Milano, Mario Cannataro

**Affiliations:** 1 Department of Law, Economics and Social Sciences, University Magna Græcia of Catanzaro, 88100 Catanzaro, Italy; 2 Data Analytics Research Center, University Magna Græcia of Catanzaro, 88100 Catanzaro, Italy; 3 Department of Medical and Clinical Surgery, University Magna Græcia of Catanzaro, 88100 Catanzaro, Italy

**Keywords:** data mining, unsupervised learning, clustering, microarrays, SNPs, DEGs

## Abstract

Gene expression and SNPs data hold great potential for a new understanding of disease prognosis, drug sensitivity, and toxicity evaluations. Cluster analysis is used to analyze data that do not contain any specific subgroups. The goal is to use the data itself to recognize meaningful and informative subgroups. In addition, cluster investigation helps data reduction purposes, exposes hidden patterns, and generates hypotheses regarding the relationship between genes and phenotypes. Cluster analysis could also be used to identify bio-markers and yield computational predictive models. The methods used to analyze microarrays data can profoundly influence the interpretation of the results. Therefore, a basic understanding of these computational tools is necessary for optimal experimental design and meaningful data analysis. This manuscript provides an analysis protocol to effectively analyze gene expression data sets through the K-means and DBSCAN algorithms. The general protocol enables analyzing omics data to identify subsets of features with low redundancy and high robustness, speeding up the identification of new bio-markers through pathway enrichment analysis. In addition, to demonstrate the effectiveness of our clustering analysis protocol, we analyze a real data set from the GEO database. Finally, the manuscript provides some best practice and tips to overcome some issues in the analysis of omics data sets through unsupervised learning.

## 1. Introduction

DNA microarrays, including Single Nucleotide Polymorphisms (SNPs) and differential expressed genes (DEGs), are becoming essential tools in omics research such as pharmacogenomics [1] and drug toxicity prediction [2]. Generally, DNA microarrays [3] are used to detect anomalies in case-control studies [4,5,6]. SNPs data [7] provide information about regions in the genome that show differences between individuals at a single base pair site. In comparison, DEGs data give information about the expression of genes responsible for the phenotype abnormalities under investigation. An omics platform, e.g., microarrays, consists of many DNA fragments spotted (or featured) onto a solid surface made of glass, silicon, or plastic. The DNA in each spot is typically called a DNA probe, and these probes usually range in size from 100 to 1,000,000 base pairs. A DNA microarray relies on the hybridization process, the hybridization between the samples of DNA representing a gene that will bind to the spot containing its complementary DNA sequence. The hybridization product, e.g., mRNA, is measured and stored as an image file for further analysis. The human genome contains around 3 billion base pairs; DNA microarrays would require many spots to cover the entire genome comprehensively. Thus, the number of probes on the microarray is generally correlated with the accuracy to represent and analyze an entire genome named microarray resolution. Microarray resolution is responsible for the microarrays’ capability to produce a large amount of data, making it challenging to interpret, even by exploiting modern computational and analytical tools [8,9,10,11,12,13,14].

To help researchers with analyzing and understanding the considerable amount of available microarray data before it becomes obsolete, several bio-statistical [15,16,17,18,19,20,21], machine learning [22,23,24,25,26,27,28], and statistical [29,30,31,32] methods are used to interpret the results’ biological meaning better. Unsupervised learning [33,34,35,36,37] techniques have been widely applied in the analysis of microarray studies to reveal hidden patterns in the data [38,39,40,41,42]. In unsupervised learning, only the input examples are provided to the model, without any expected output, to make it learn some hidden structure in the input data independently. Clustering methodology belongs to the unsupervised learning technique. Clustering recognizes the most common elements around which to group similar elements, to reduce the data necessary for understanding a phenomenon, which is a very important aspect in the analysis of omics microarrays data.

In mathematical terms, the concept of similarity is translated in distance. The calculation of similarity becomes that of distance in the space of *n*-dimensions, where *n* indicates the number of variables in which the data are described. Many statistical and computational approaches, including hierarchical, partitive, and density-based, are available for clustering. While these algorithms are comparable in terms of performance (i.e., there is no prevalence of one method over the others), there are multiple issues and challenges due to the computational analysis of microarray data.

The task of microarray data analysis differs from other data analysis in that many proven feature dependencies already exist in microarray data, resulting in a high degree of redundancy. Thus, by exploiting clustering techniques, it is possible to identify subsets of features with low redundancy and high robustness, speeding up the identification of new bio-markers.

Clustering involves projecting the data in a low-dimensional space to explore the relationships among tested samples for visual inspection and exploratory analyses. Clustering techniques group the experimental samples based on their response across the transcriptomics profile. Indeed, in biomedical research, clustering approaches group together objects, e.g., genes and tissues more similar than those in different clusters. At the same time, it is useful for dimensionality reduction purposes, playing a crucial role in pattern and knowledge recognition. This is especially true in high-throughput omics technologies that are contributing to provide an increasing number of publicly available massive omics data sets composed from different biological variables for the same samples, allowing the integration of complementary information and bringing a deeper insight into the same biological processes. For example, clustering methods can be utilized to analyze toxicity prognosis experiments to identify drugs showing similar mechanisms of action in a large cohort of patients. In the medical field, they can be used to identify groups of patients who respond differently to medical treatments. Although cluster analysis approaches are robust, great care must be taken in applying these approaches. Even though the algorithms are well characterized and reproducible, the wrong choice concerning the selected normalization method or metrics will place different objects into distinct clusters and could bias the results. For instance, unrelated clustering data will still produce clusters, although they might not be biologically meaningful. Thus, the challenge is selecting, handling, arranging the data and applying the algorithms appropriately so that the models that arise from the data are biologically relevant. The choice of the most suitable method to analyze microarray data sets is related to the high degree of redundancy that exists in microarray data sets. In this regard, we propose a general protocol to analyze DEG data sets to identify subsets of features with low redundancy and high robustness by using K-Means and DBSCAN, speeding up the identification of new bio-markers through pathway enrichment analysis. Finally, to prove the effectiveness of the presented clustering analysis protocol, we downloaded a real data set from the GEO database that we analyzed by using K-Means and DBSCAN. The dataset, the Python code to reproduce to results of this work and the example results are available at https://github.com/mmilano87/PCAPxDEG (accessed on 3 October 2022). The rest of the manuscript is arranged as follows. Section 2 describes the type of omics data, e.g., SNP and DEGs data sets, and the principal preprocessing methodologies. Section 3 introduces some clustering metrics highlighting the most suitable for handling DEGs data. Section 4 presents the main steps of K-Means and DBSCAN algorithms. Section 5 introduces a general analysis protocol based on K-Means and DBSCAN to deal with DEGs data sets obtained by using omics methodology. Section 6 discusses the obtained results, providing some best pratices to improve the analysis of DEGs data through clustering. Finally, Section 7 concludes the manuscript.

## 2. Materials and Methods

### 2.1. Microarray Raw Data

Gene expression and SNP data assessed by microarrays are preprocessed using image analysis techniques to extract expression values and SNPs from images, making expression values and SNPs comparable across microarrays. The preprocessing is completed using a specific microarray platform software provided by the vendor. The expression data are commonly represented as a matrix of values where each column indicates a sample, and each row denotes a probe (i.e., a specific gene). The cell (i,j) contains the expression level of gene *i* evaluated in sample *j*. Table 1 shows a simple gene expression data matrix.

Conversely, SNP data are represented as a string matrix where each column indicates a sample, and each row denotes a probe (i.e., a specific gene). The string contained in the cell (i,j) is the detected SNP on gene *i* related to sample *j*. DNA’s bases are adenine (A), cytosine (C), guanine (G), and thymine (T), which define the SNP alphabet. Given the following two sequences [AGTGA] and [AGCGA] belonging to two different subjects, we have an SNP into the 3rd position denoted as T/C. Table 2 shows a simple SNP data matrix.

Knowing how microarray data are arranged is essential to define the proper data learning strategy and how the results should be interpreted [43]. The gene expression data matrix is already in a suitable format to be used as input for the majority of clustering algorithms. The only recommended operation to complete for speeding up the analysis is filtering out rows, e.g., genes that are not expressed or are the same across all the subjects. Conversely, the SNP data matrix is not in a suitable format to perform clustering analysis. Before performing SNP data clustering analysis, the literal symbols must be mapped into discrete numerical representation using feature transformation [44,45]. This is mandatory, as the existing clustering approaches can only deal with numerical vectors but not string vectors. Several approaches are available to convert SNP literals into numerical vectors [46,47,48].

### 2.2. Microarray Data Filtering

Microarray data filtering [49] reduces the size of both matrices, e.g., gene expression and SNP matrices. Gene expression data filtering removes genes that are not expressed or do not exhibit variation across the subjects. The filtering methodology removes the genes not expressed or indefinite for the following analysis. The gene filtering strategy includes a threshold of the gene variance across the matrix, removing all the genes whose expression is below the chosen threshold value. SNP data filtering removes all the genes that do not show variation across the samples. Usually, the SNP filtering strategy includes a threshold of the SNP occurrences across the matrix, removing all the rows whose occurrences are below the chosen threshold percentage value.

### 2.3. Microarray Data Normalization

Although clustering methods can be applied to the raw data, it is often more helpful to forego the analysis by normalizing (or homogenizing) the expression values [50]. Values normalization allows mitigating the possible bias due to the distance or similarity measure. Cluster analysis depends on a distance or similarity measure. Because distance or similarity measures are sensitive to differences in the absolute expression values scale, microarray data for clustering often need to be transformed to adapt to different scales.

## 3. Clustering Metrics

Many metrics are available to compare patterns from microarray data. However, the metric to use for group discovery will impact the results. Distance and similarity are the most used metrics in unsupervised learning. In mathematical terms, the concept of similarity is translated in distance. The similarity calculation becomes the distance in the space of *n*-dimensions, where *n* indicates the number of variables described in the data. The distance evaluates how far apart two points p and q are in the space. For a distance measure d(·) to be a valid measure, it has to always satisfy the following conditions:

1. d(·) is always non-negative;
2. d(·) is symmetric;
3. d(·) must satisfy the triangle inequality;
4. d(·) must be zero only when evaluating the distance of a point from itself.

Without losing in generality, distance d(·) assesses how far or close pairs of points are, e.g., gene expressions or SNPs data. Well-known distance metrics are Euclidean [51], Manhattan [52], and Chebychev [53] distance, which are suitable to analyze gene expressions data.

- The Euclidean distance evaluates the distance between each pair of points. Equation (1) reports the Euclidean distance definition.
  (1) $$e\left(x,y\right)=\sqrt{\sum_{i=1}^{n}{\left|\left(x_{i}-y_{i}\right)\right|}^{2}}$$
  The Euclidean distance is computed by subtracting the value of gene *i* from gene *j*, squaring it in each sample, and then taking the square root of the sum. Euclidean distance is suitable for handling low-dimensional data, and when measuring the vectors’ magnitude is not necessary.
- The Manhattan distance m(·) between two points is equal to the norm of the distance between two points $p_{1}$ and $p_{2}$ in a plane with coordinates $p_{1}=\left(x_{1},y_{1}\right)$ and $p_{2}=(x_{2},y_{2})$, it is $m=|x_{1}-x_{2}|+|y_{1}-y_{2}|$. Equation (3) reports the Manhattan distance definition. Manhattan distance is easily generalized to higher dimensions. Given an array of *n* coordinates, the m(·) provides the differences between any pairs of coordinates. Manhattan distance is suitable to manage attributes containing discrete or binary attributes.
  (2) $$m\left(x,y\right)=\sum_{i=1}^{n}\left|\left(x_{i}-y_{i}\right)\right|$$
- Chebyshev distance ch(·) measures the maximum difference between the distinct points pairs of two arrays, which is also known as maximum value or chessboard distance. Chebyshev distance is suitable to deal with ordinal or quantitative data sets. Chebyshev distance is defined in Equation (3).
  (3) $$ch\left(x,y\right)=\max_{i}\left|\left(x_{i}-y_{i}\right)\right|$$

Conversely, the similarity function tries to quantify how “similar” two data points are from the distance function. It must be maximized because the more similar the two points are, the closer they are; that is, they belong to the same cluster.

A similarity metric s(·) must hold the following criteria:

1. s(·) is symmetric;
2. s(·) fulfills the triangle inequality;
3. s(·) between the same data point must be non-negative;
4. s(·) between any two different data points cannot be larger than the similarity between a data point and itself.

Well-Known similarity metrics encompass Cosine [54], Jaccard [55], and Sorensen–Dice [56].

- Cosine metric computes similarity converting two vectors in points and estimating the cosine of the angle between the two points in the vectors’ space.
  (4) $$c\left(v,w\right)=\cos\Theta =\frac{v\cdot w}{\left\|v\|w\right\|}$$
  A limit of cosine similarity measure is the impossibility of evaluating the magnitude of the vectors, it can only measure their direction. Cosine similarity is used for handling high-dimensional data sets and when it is not necessary to evaluate the magnitude of the vectors.
- Jaccard index computes the similarity and diversity by estimating the ratio between the size of the intersection between two groups divided by the size of the union of two groups. Thus, it is also known as intersection over union.
  (5) $$J\left(v,w\right)=1-\frac{v\cap w}{v\cup w}$$
  Jaccard index is used to analyze data sets containing binary or binarized data.
- The Sorensen–Dice (SD) index computes the similarity and diversity by estimating the percentage of overlap between two groups. The SD index is used to analyze data sets containing binary or binarized data.
  (6) $$SD\left(v,w\right)=\frac{2|v\cap w|}{\left|v|+|w\right|}$$

## 4. Clustering Algorithms

Clustering is an unsupervised procedure to organize data into groups of items with similar patterns distinctive for each group. In microarray data analysis, the main goal of the clustering procedure is to organize genes or samples with common characteristics or profiles together. Collecting genes or samples together allows researchers to make meaningful biological assumptions about the set of gene or sample groups. Clustering procedures can be classified as hierarchical or non-hierarchical. Hierarchical clustering groups objects into clusters and specifies relationships among items in the clusters. Conversely, non-hierarchical methods group items into clusters without establishing relationships between objects in the same cluster. Hierarchical clustering may be divided into two categories: agglomerative and divisive. Agglomerative clustering starts with the hypothesis that each object is a cluster, grouping similar objects into bigger clusters (bottom–up strategy). Conversely, divisive clustering begins with all the objects in one cluster. Next, it breaks the big cluster into smaller clusters of objects with comparable properties. Hierarchical agglomerative clustering considers each object a cluster. It is an iterative methodology where the objects are consecutively grouped until all the objects are not included. The main steps of the method are the following. The first step calculates the pairwise distance between the objects to group. Based on the pairwise distances between objects, objects that are similar among them are grouped into clusters. Next, pairwise distances between the clusters are re-calculated, and similar clusters are grouped iteratively until all the objects are included in a single cluster. The output of the hierarchical agglomerative approach can be represented as a dendrogram, where the distance from the branch point indicates the distance between the two clusters of objects. Hierarchical divisive clustering considers the entire set of objects as a single cluster, and iteratively, the cluster is broken down into clusters with similar patterns (top–down strategy). The hierarchical divisive clustering considers each cluster separately, and the divisive process is repeated until all objects have been separated into single objects.

The main goal of both variants is to reach through the consecutive partitioning the optimal data separation.

Conversely, from hierarchical clustering, non-hierarchical clustering groups existing objects into some predefined clusters rather than organizing them into a hierarchical structure. Non-hierarchical clustering requires predetermination of the number of clusters.

Another category of cluster algorithms is based on the concept of density and does not require any assumptions about the formation of the classes. The intuitive concept of density starts from the consideration that a cluster in the space of a point is a region of high point density. Thus, each pattern of a cluster must have at least a certain number of other patterns in its neighbor, so the density in the neighborhood of the considered pattern must exceed a certain threshold.

Gene clustering of microarray data has been widely used to group and organize DEGs and SNPs. Gene clustering is also used for multi-group data in which several gene expression patterns may exist. Thus, identifying gene expression patterns and grouping genes into expression groups could provide much greater insight into their biological processes.

In the following, we provide a brief description of two of the most known divisive and density cluster algorithms, K-Means and DBSCAN.

### 4.1. K-Means

K-Means [57] is a popular non-hierarchical clustering method. The K-Means algorithm belongs to the category of clustering based on prototypes. Clustering based on prototypes means that each cluster is represented by a prototype, e.g., centroid (the mean), of similar points and with continuous characteristics, or the medoid (the points that occur most frequently) in the case of categorical features. K-Means is very effective in identifying clusters of spherical shape. One of its weaknesses is that it requires specifying a priori the number of clusters, *k*. An inappropriate choice of *k* can result in poor clustering arrangement. K-Means performs the following four steps.

1. Randomly selects *k* centroids of the sample points, which will act as interim cluster centers. The number of clusters can be chosen randomly or estimated by first performing hierarchical clustering of the data. Next, each cluster is initialized by computing its centroid.
2. Assign the closest centroid to each sample. Individual objects are moved from one cluster to another depending on which centroid is closer to the object.
3. Move centroids to the center of the samples that have been assigned to it. This procedure of calculating the centroid for each cluster and re-grouping objects closer to available centroids is performed in an iterative manner for a fixed number of times or until convergence (state when the composition of clusters remains unaltered by further iterations). However, there is no guarantee that the procedure will converge.
4. Repeat steps 2 and 3 until the cluster assignment stops changing or when a certain tolerance or a maximum number of iterations is reached.

### 4.2. DBSCAN

DBSCAN (Density-Based Spatial Clustering of Applications with Noise) [58] defines the concept of density as the number of points that fall within a given radius ε. In DBSCAN, a specific label is assigned to each object (point) using the following criteria.

1. A point is considered a core point if at least a certain number (MinPts) of neighboring points fall within the specified radius.
2. A boundary point is a point with fewer neighbors than MinPts that they are within but still comes within the radius of distance from a core point.
3. All other points that are neither core nor boundary are assumed to be noise points.

After labeling the points as core, boundary, or noise, the DBSCAN’s core algorithm can be summarized in two simple steps.

1. Create a distinct cluster for each core point or connected group of core points (core points are connected if they are no more than ε apart from each other).
2. Assign each boundary point to the corresponding core point cluster.

## 5. Gene Expression Clustering Analysis Protocol

This section defines a general analysis protocol for analyzing massive gene expression data sets through unsupervised learning methods. First, we introduce the most suitable preprocessing methods for the gene expression data, which is followed by the dimensionality reduction and feature scaling. Next, we show how to tune K-Means and DBSCAN to produce relevant clusters exploiting the handled data. Finally, as a possible use case, gene clusters can be used to perform pathway enrichment analysis, linking each gene with the affected underlying biological mechanisms.

### 5.1. Synthetic Gene Expression Data Set

To perform the analysis, we generated a synthetic gene expression microarray data set obtained exploiting the features of the Affymetrix Human Genome U133 Plus 2.0 Array. The GeneChip Human Genome U133 Plus 2.0 Array comprehensively analyzes genome-wide expression on a single array. It provides comprehensive coverage of the transcribed human genome on a single array, analyzing the expression level of over 47,000 transcripts and variants, including well-characterized human genes. The generated data set contains 1000 samples (columns) and 20,531 probes (rows) for each sample connected to the detected gene expression levels.

### 5.2. GEO Breast Cancer Data Set

Gene expression Omnibus (GEO) [59,60] is a public repository of high-throughput functional genomics data submitted by the research community. From GEO, we downloaded the *GSE183947* data set. The *GSE183947* data set refers to breast cancer and contains 30 pairs of normal and cancerous tissues from the same excision that were investigated using the RNA sequencing (RNAseq) GPL11154 Illumina HiSeq 2000 (Homo sapiens) platforms and collected from the Affiliated Cancer Hospital of Guangzhou Medical University. The *GSE183947* data set contains 20,246 rows, e.g., the platform’s genes and 61 columns, e.g., tissues. More details about the *GSE183947* data set are available at: https://www.ncbi.nlm.nih.gov/geo/query/acc.cgi?acc=GSE183947 (accessed on 3 October 2022).

### 5.3. Clustering Analysis Protocol

Understanding how to correctly perform clustering algorithms using gene expression data is fundamental for partitioning data into groups or clusters that can help to identify meaningful knowledge for the domain expert.

The task of gene expression data analysis differs from other data analysis in that many proven feature dependencies already exist in gene expression data, resulting in a high degree of redundancy. Thus, by exploiting clustering techniques, it is possible to identify subsets of features with low redundancy and high robustness, speeding up the identification of new bio-markers.

**Figure 1:**
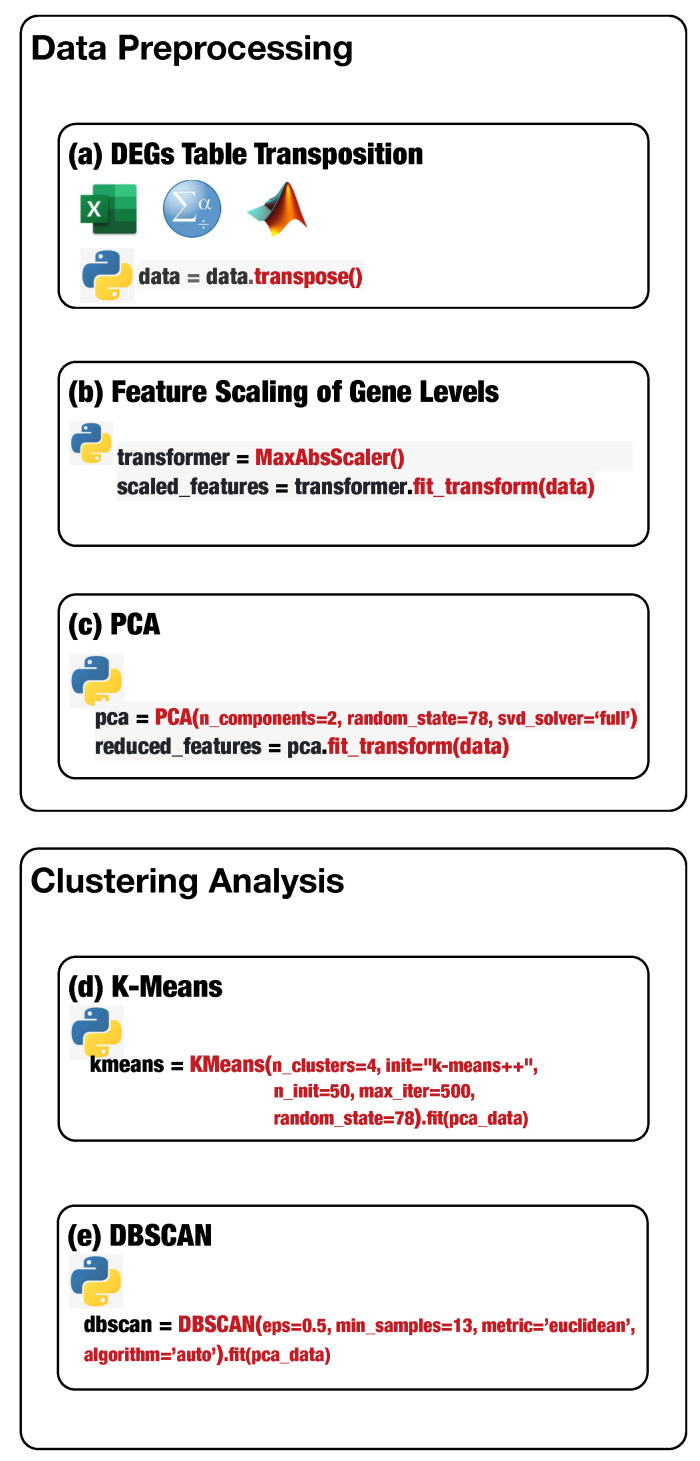
The Python statements to perform the main steps of data preprocessing and run the DEGs data analysis through K-Means and DBSCAN algorithms.

Hence, we present a general clustering protocol for large differential gene expression data sets commonly derived from genome-scale (omics) technology. The protocol is intended for experimental biologists, researchers without any programming skills, and bioinformaticians interested in making the interpretation of their omics data productive. A detailed description follows of a general step-by-step protocol using the K-Means or DBSCAN algorithm available in the *scikit-learn Python package*. Figure 1 shows the main steps of the proposed analysis protocol.

Omics-scale experiments yield raw data that must be processed to obtain gene-level information suitable for clustering analysis (independently from the chosen algorithm). Gene expression data sets are organized as huge tables where the rows contain the probes, e.g., the DNA spots related to a specific gene, whereas the columns have the samples (as depicted in Table 1). A generic cell, i.e., (i,j), contains the amount of hybridization (e.g., the level of expression) referring to the gene *i*-th and the sample *j*-th. In this form, gene expression data sets are unsuitable to be handled by the K-Means and DBSCAN algorithms.

Preprocessing is a fundamental step for putting data in a suitable format for K-Means and DBSCAN. In particular, it is necessary to perform the *transposition* of the input data set, because data arrangement significantly affects the K-Means and DBSCAN performance and the result’s relevance. Transposition consists of switching rows with columns, making data in a suitable format for K-Means and DBSCAN to provide relevant gene groups. The transposition step can be performed straightforwardly by loading the input data set in Excel, Matlab, or SPSS using the available automatic function *transpose* or using the Python *transpose* method (Figure 1a defines the Python statement to perform table transposition on the loaded DEG data) available in *pandas* and *numpy* libraries.

Next, the detected DEGs values present higher variability, contributing to some bias because machine learning algorithms consider higher values more relevant than the lower ones. Thus, it is mandatory to transform all the gene expression values to the same scale. The technique of transforming numerical features to the same scale is known as *feature scaling*. Feature scaling is necessary, especially for distance-based machine learning algorithms, because it can significantly impact the algorithm’s performance. Feature scaling can be completed through the *MaxAbsScaler* estimator available in the sklearn.preprocessing package (Figure 1b defines the Python statement to instantiate and run a scaler object). MaxAbsScaler scales and translates each element individually such that the maximal absolute value of each element in the training set will be 1.0. It does not shift/center the data and thus does not destroy any sparsity, making *MaxAbsScaler* suitable for scaling gene expression data.

The high number of features (e.g., the number of genes equals to 20,532) may negatively impact the final results; consequently, feature reduction is mandatory. Unbiased feature reduction can be achieved using statistical techniques such as principal component analysis (PCA). PCA reduces the number of features by either removing or combining them. PCA available in scikit-learn decomposes a multivariate data set in a set of successive orthogonal components that present a maximum amount of variance. Figure 1c shows the Python statement to instantiate and run a PCA object.

Finally, DEG data are in the optimal format for the K-Means and DBSCAN algorithms.

**Figure 2:**
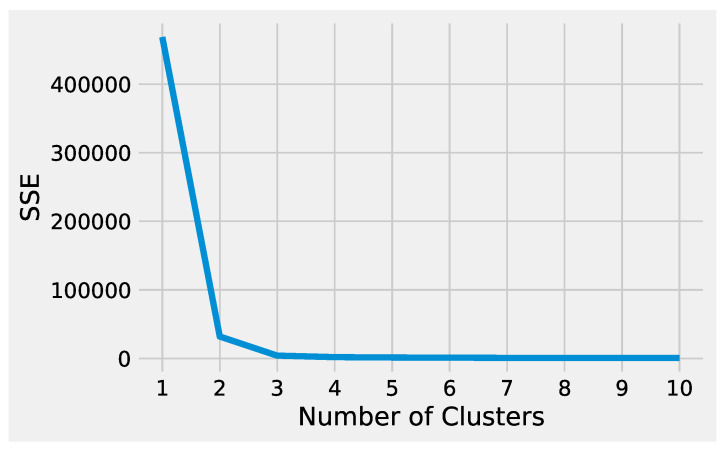
The SSE values obtained from K-Means varying the number of clusters *k*.

First, we describe how to set up K-Means to effectively analyze the preprocessed input data. Next, we delineate how to tune DBSCAN to handle the preprocessed input data, highlighting the difference between the two methods. Before starting the K-Means algorithm, it is necessary to tune some parameters. The most crucial K-Means parameter to tune is the number of clusters *k*. The optimal number of clusters *k* could be identified by evaluating the *sum of the squared error (SSE),* because the goal of K-Means is to minimize the SSE. Figure 2 illustrates the obtained SSE values varying *k* into the range {1,…,10}. In Figure 2, the point where the curve starts to bend, known as the *elbow point*, represents an adequate trade-off between error and the number of clusters achieved for k=3.

**Figure 3:**
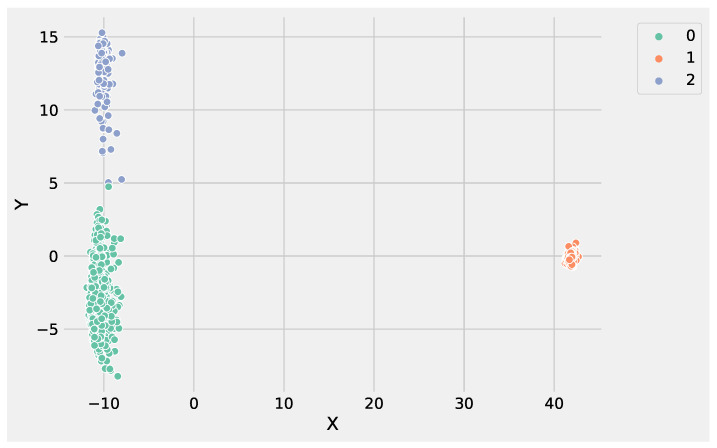
The clusters obtained by K-Means using the input gene expression data set. In the figure, the *X* label refers to the gene expression values, whereas the *Y* label indicates the predicted clusters.

The other K-Means parameters to tune are: the *initialization technique* parameter, which must be set up using the option k-means++ to ensure efficient centroids initialization and to boost the convergence; the *number of initializations* to perform; and the *maximum number of iterations* for each initialization. Figure 1d conveys the Python statement to instantiate and run a K-Means object on the preprocessed DEG data. Finally, the obtained clusters can be visualized, and the genes for each cluster can be stored in a file for subsequent analysis. Figure 3 shows the obtained groups performing K-Means using the input data set.

**Figure 4:**
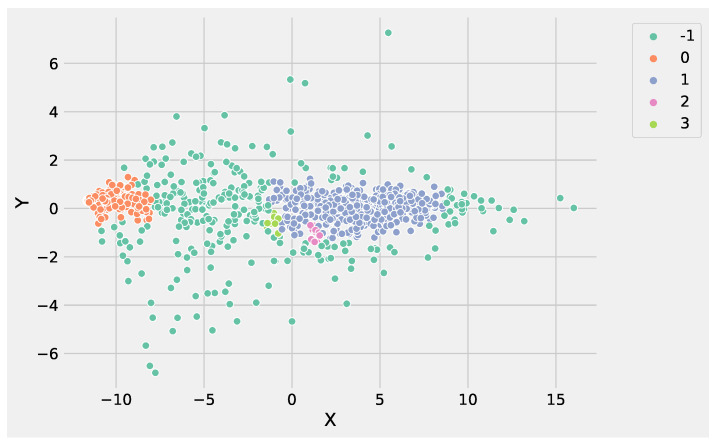
The clusters obtained by DBSCAN using the input gene expression data set. In the figure, the *X* label refers to the gene expression values, whereas the *Y* label indicates the predicted clusters.

DBSCAN, unlike K-Means, does not need to define the clusters number to group data; instead, it needs to tune min_samples and eps parameters allowing to define the size of the density regions. If eps is chosen to be too small, some data could be not clustered and labeled as noise through the −1 value. Instead, if eps is too large, close clusters could be merged into one cluster, eventually producing a single cluster. The parameter min_samples controls the number of samples (or total weight) in a neighborhood for a point to be considered as a core point. The other parameters to tune are the following. The metric parameter is used to calculate the distance between instances in a feature array; we set the metric using the Euclidean distance, e.g., metric=’euclidean’. The algorithm parameter defines the *NearestNeighbors* module to compute point-wise distances and find nearest neighbors. The method should be chosen considering the nature of the problem; in this case, we set the algorithm using auto, e.g., algorithm=’auto’. Figure 4 illustrates the identified groups performing DBSCAN using the input data set.

Analyzing Figure 3 and Figure 4, it is worthwhile to note that DBSCAN produced four not well-separated clusters, and at the same time, a huge number of points were labeled as as noise, e.g., identified from the label −1. These results highlight the difficulty of DBSCAN to deal with this DEG data set. Conversely, K-Means was able to produce three well-separated clusters and was more informative than DBSCAN. As a result, the genes’ groups yielded from K-Means and DBSCAN can be employed to perform pathway enrichment analysis.

Pathway enrichment analysis searches for pathways whose genes are enriched from the list of genes of interest, e.g., the group of genes obtained using K-Means. Pathway enrichment analysis is a statistical method to link a gene list of interest with the affected biological mechanisms [61]. The default pathway enrichment analysis implemented in BiP [15], cPEA [16] and PathDIP [62] software tools searches for pathways whose genes are particularly enriched (i.e., over-represented) in the fixed list of genes of interest with respect to genes randomly chosen. The *p*-value of the enrichment of a pathway in BiP is computed using a hypergeometric test, and multiple-test correction is applied. Table 3 shows the enriched pathways from BiP by using the gene belonging to *cluster1*.

Pathway enrichment analysis of omics data has several advantages compared to the analysis of single genes. First, it provides information about genes’ interaction in form of an interaction system or pathway. Second, it simplifies the result interpretation by providing the name of the affected biological mechanisms, e.g., *extracellular matrix organization*, and finally, it can speed up the identification of new bio-markers and drug targets.

### 5.4. GSE183947 Data Set Analysis

To validate the proposed Python clustering analysis protocol, we analyze the GSE183947 data set by using the K-Means and DBSCAN algorithms available in the sklearn.cluster library.

The K-Means results have been obtained tuning its main parameters as follows: kmeans=KMeans(n_clusters=3, init="k-means++", n_init=50, max_iter=500, random_state=42).fit(pca).

The DBSCAN results have been obtained tuning its main parameters as follows: dbscan = DBSCAN(eps=0.3, min_samples=10, metric=’euclidean’, algorithm=’auto’).fit(pca).

**Figure 5:**
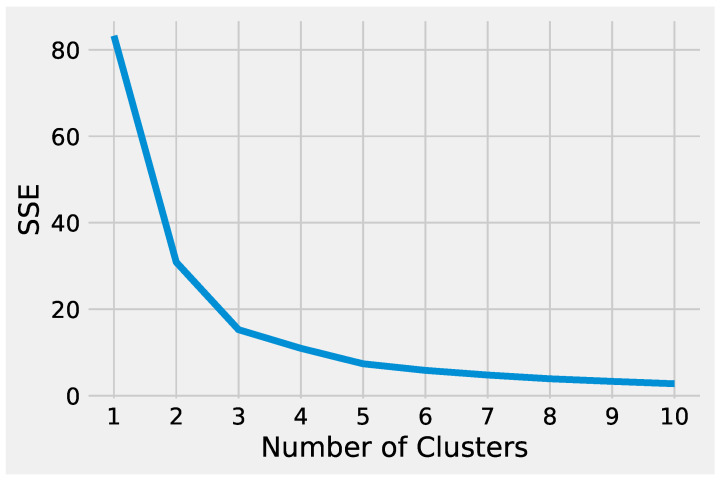
The SSE values obtained from K-Means varying the number of clusters *k*.

Analyzing the results produced by K-Means and DBSCAN, it is worthwhile to note that both methods identified two groups. In particular, the value used to set the parameter *k* of K-Means was obtained evaluating the SSE, as shown in Figure 5. DBSCAN needs to tune epsilon and the minimum number of points to define the regions’ diameter and the core points without it being necessary to define the number of clusters. The main difference between the K-Means and DBSCAN results concerns the different number of points in the two groups. K-Means assigns only a point to *cluster 1*, e.g., the gene *ATP5H*. Whereas DBSCAN labeled six genes as noise, e.g., belonging to *cluster-1*: *NEDD4, BLOC1S6, WDR74, KIAA1328, ATP5H* and *NPIPA7*.

The genes belonging to the two clusters can be used to perform pathway enrichment analysis, making it possible to link genes with the affected underlying cellular mechanisms. To perform pathway enrichment, we use BiP and the Homosapiens pathway from the Reactome database. The enrichment analysis using the six genes belonging to *cluster 1* did not enrich any pathway. In contrast, performing pathway enrichment by using the genes within the *cluster 0* computed from both methods, it was possible to enrich several biological pathways. Table 4 reports for space reasons only 10 enriched pathways that are the same for both gene lists. All the enrichment results are available at: https://github.com/mmilano87/PCAPxDEG/tree/main/results (accessed on 3 October 2022).

**Figure 6:**
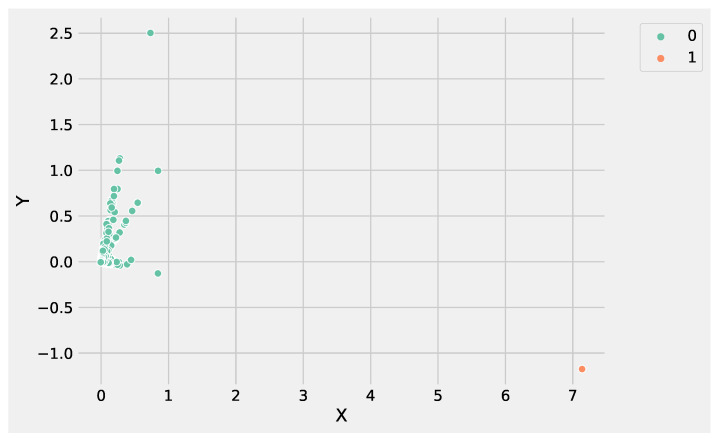
The clusters obtained by K-Means using the GSE183947 RNASeq expression data set. In the figure, the *X* label refers to the gene expression values, whereas the *Y* label indicates the predicted clusters.

**Figure 7:**
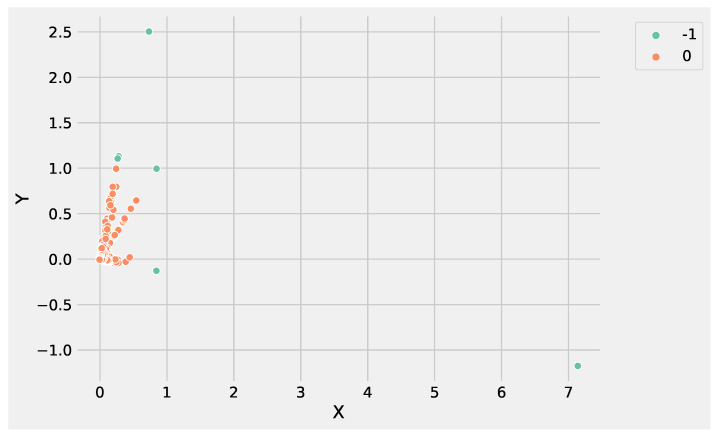
The clusters obtained by DBSCAN using the GSE183947 RNASeq expression data set. In the figure, the *X* label refers to the gene expression values, whereas the *Y* label indicates the predicted clusters.

Figure 6 and Figure 7 show the clusters produced using K-Means and DBSCAN, analyzing the GSE183947 data set. Both methods grouped the DEGs in two clusters, with the difference that DBSCAN clustered some points as noise that were close to the core region. Conversely, K-Means identified the only outlier, e.g., the farthest point with coordinates (−0.9;7.1).

The reason is that K-Means updates the centroids at each iteration, making it possible to rearrange the centroid value in order to expand cluster density by adding the new points, making K-Means more suitable to deal with data sets with a high degree of redundancy. Conversely, DBSCAN core points identification is based on the ϵ and the minimum number of points that are fixed, and it is not possible to re-arrange at each new iteration. In addition, the high degree of redundancy of DEG data sets affects the border points identification, contributing to split data instead of expanding the cluster density.

## 6. Discussion

The proposed step-by-step protocol describes a general methodology to analyze massive DEG data sets using K-Means and DBSCAN, combining suitable Python statements as lego pieces without requiring advanced computational skills. For each protocol step, we identified and used the most appropriate analytical methods and their implementation, such as Python’s class and functions, to optimally deal with DEG’s data sets. In particular, the data normalization is performed through the *MaxAbsScaler* class, which is a method that does not affect the data distribution, avoiding losing information on gene expression levels. In addition, the high degree of redundancy of the DEG data sets is handled through the *PCA* class that centers but does not scale the input data for each feature before applying the Singular Value Decomposition (SVD). At the end of the preprocessing steps, the data are suitable to feed K-Means or DBSCAN for analyzing data aiming to group and stratify similar genes related to the biological question of interest (e.g., tumor versus normal, treated versus untreated). In this way, it is possible to identify drugs showing similar mechanisms of action in a large cohort of patients, identifying groups of patients’ genes that are responsible for the different responses to medical treatments.

It is worthwhile to note that the proposed analysis protocol is general and could be used to investigate any type of numerical data sets arranged in a tabular format, and it is not only limited to analysis of DEGs data. Moreover, keeping in mind the lego analogy, this protocol can be easily improved even for exploiting high-performance hardware just by using the high-performance version of K-Means available in the PySpark library. To extend the script to exploit performance computing, it is necessary to only import the PySpark library using the following statement (see Listing 1),

**Listing 1.** The required statement to link into the script the PySpark package.

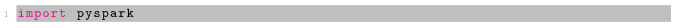



Then, we use the K-Means algorithm available in the PySpark library, using the instruction in Listing 2.

**Listing 2.** The statements necessary to link into the script the PySpark package.

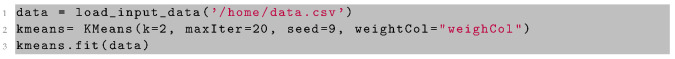



Appendix A describes in a very detailed way all the Python statements composing the analysis protocol.

Now, we will further validate the contribution of clustering analysis in helping researchers discover subgroups of DEGs responsible for the onset and progression of complex diseases, which could be used as new possible bio-markers to define new treatment strategies based on the individual genome. To support this idea, we performed pathway enrichment analysis by individually using the grouped genes produced from K-Means and DBSCAN from the GSE183947 data set. As a result, it was possible to enrich the same pathway by using the DEGs belonging to the K-Means and DBSCAN clusters. Table 3 reports 10 enriched pathways. We manually browsed the literature to seek evidence for linking the enriched biological pathways with breast cancer. The pathway with index *(1) Metabolism of proteins*, *(2) Signaling pathways (3) Gene expression (transcription)* and *(6) Metabolism* from Table 3 are crosstalk pathways playing a critical role in breast cancer as described in [63,64]. The *(4) RNA polymerase II transcription* pathway performs a regulating function in breast cancer as reported in [65]. The role of pathway *(7) Post-translational protein modification* is discussed in [66]. The pathways *(8) Cellular responses to stimuli* silencing prion protein in MDA-MB-435 breast cancer, as reported in [67], and *(9) Cellular responses to stress* regulate the MCF-7 breast cancer cells entering quiescence [68]. The role of the pathway *(5) Immune system* in breast cancer is discussed in  [69]. Finally, Furth et al. [70] describes the involvement of the *developmental biology* pathway in breast cancer through the signaling pathway, highlighting the cross-relation between pathways (1) and (10) of Table 3.

Even using the most suitable statements and tuning properly the main K-Means and DBSCAN parameters, there are other factors to take into account to ensure high accuracy of the results and reproducibility. Among them, it is worthy to keep the following in mind: *(i)* clustering analysis depends on gene sets and databases used in the investigation, and outdated resources strongly impact the study quality and results. *(ii)* Genes are associated with many diverse identifiers. The recommendation is to use the unambiguous, unique, and stable identifier, as some identifiers become obsolete over time. We recommend using data sets based on the Entrez Gene database, or gene symbols, for human genes. Because gene symbols change over time. As a final tip, the recommendation is to maintain both gene symbols and Entrez Gene IDs because there are many tools available that support the automatic conversion of multiple identifier types to standard identifiers.

In addition to the programming tips, we provide some useful tips concerning the advantages and limitations about the choice of the available cluster algorithm and metrics. The provided tips could be exploited in case users want to further customize the analysis protocol by using a different either cluster algorithm or metric to evaluate their data, or use different unsupervised algorithms for evaluating which best addresses the objectives that the study intends to achieve. Indeed, the possibility of gene expression data sets being analyzed through many clustering models could allow highlighting polymorphic variants and selecting patient subsets for constructing personalized classifiers, making them a promising tool for personalized prescription for cancer patients [2].

The main limitations of the listed metrics follow. The cosine similarity does not take into account the difference in rating scale between different points or vectors. In addition, cosine similarity computed the difference between the two points or vectors in terms of directions and not magnitude. A major disadvantage of the Jaccard index is that it is highly influenced by the size of the data sets. Large data sets can have a huge impact on the index, as it could significantly increase the union while keeping the intersection similar. In contrast, the Sorensen–Dice metric weights each entity inversely proportionally to the size of the relevant group rather than treating them equally. It is worth nothing that the Sorensen–Dice dissimilarity measure is not a metric, since it does not satisfy the triangle inequality condition. The Euclidean distance is the length of the line segment joining a given pair of points in a grid/graph. The Euclidean distance only makes sense when all the dimensions have the same units (such as meters), since it involves adding the squared value of them. Manhattan distance calculates the distance between two data points in a grid as a path. The Manhattan distance is the sum of the absolute values of the elements of a vector. Manhattan drops points with offsets in another dimension, making it suitable to deal with high-dimensional data.

One potential problem with many hierarchical clustering methods is that as clusters grow, the centroid that represents the cluster might no longer describe any of the items in the cluster (e.g., genes). Conversely, divisive approaches are not affected by this limitation, because they partition data (e.g., genes or SNPs) into groups with similar patterns.

The main advantage of the DBSCAN is that it can cluster data with different distributions. However, it is worth noting that DBSCAN suffers from the dimensionality curse due to the increasing number of features in the data set. This problem is particularly evident using the Euclidean distance. Contrarily, K-Means has the advantage that it is scalable for large data sets. Conversely to K-Means, DBSCAN does not assume that clusters must have a spherical shape. In addition, DBSCAN differs from K-Means since it is hierarchical and does not necessarily assign each point to a cluster but can remove noise points.

One potential disadvantage of K-Means clustering is that it requires defining a priori the number of clusters, and it is sensitive to outliers. It is worth noting that changing the order of data can produce different results.

Finally, the shape of the clusters detected with any metrics varies with the data point’s scale imbalance. To avoid this problem, it is suggested to normalize the scale of each component of the data. In addition, as the data dimensionality increases, Euclidean, Manhattan, and Chebyshev distances become less helpful. This is due to the curse of dimensionality, concerning the notion that higher-dimensional space does not act as we would expect from 2D or 3D space.

## 7. Conclusions

The assumption behind the use of clustering techniques to analyze microarrays data is that genes in a cluster provide insight into the gene interactions impacting the outcome of the cellular machinery. However, the identified clusters depend on the respective methods and metric and/or normalization methods. These methods can significantly affect the outcome of any analysis and its reproducibility. The choice of the most suitable methods should be data-driven, highlighting that there is no single way to analyze data. However, different techniques might be more or less appropriate for different data sets. In particular, the case study pointed out that K-Means is more suitable than DBSCAN to handle DEGs data sets obtained from microarray and RNASeq. Furthermore, applying more than one technique to explore a particular data set could clarify other relationships between the data. In this work, we provided a simple step-by-step protocol to simplify the use of programming language to perform data preprocessing and clustering analysis even for users without programming skills. In particular, the analysis protocol highlights that by changing just one statement, e.g., the instantiation of the clustering class, it is possible to run different clustering algorithms. In this way, the identification of new bio-markers is speeding up. Finally, we provided some best practices and tips to overcome issues in the study of omics data sets through unsupervised learning.

## Figures and Tables

**Table 1:** Gene expression data matrix.

	$S_{1}$	*…*	$S_{m}$
$probe_{1}$	32.3	*…*	121.4
$probe_{2}$	87.59	*…*	95.78
⋮	⋮	⋮	⋮
$probe_{n}$	123.4	*…*	95.78

**Table 2:** SNPs data matrix.

	$S_{1}$	*…*	$S_{m}$
$probe_{1}$	G/A	*…*	C/G
$probe_{2}$	A/G	*…*	T/T
⋮	⋮	⋮	⋮
$probe_{n}$	G/G	*…*	G/A

**Table 3:** The BiP-enriched pathways using the genes belonging to cluster 1.

Enriched Pathway	*p* Value
Intra-Golgi traffic	0.0045
Glutamate Neurotransmitter Release Cycle	0.0061
Astrocytic Glutamate–Glutamine Uptake And Metabolism	0.0065
Neurotransmitter uptake and metabolism In glial cells	0.0069
Trafficking of GluR2-containing AMPA receptors	0.0072
Defective SLC24A1 causes congenital stationary night blindness 1D (CSNB1D)	0.0076
MPS VII—Sly syndrome	0.0076
Extracellular matrix organization	0.0080
Presynaptic depolarization and calcium channel opening	0.0098
The canonical retinoid cycle in rods (twilight vision)	0.0102

**Table 4:** The BiP-enriched pathways using separately the genes belonging to *cluster 0* obtained from DBSCAN and *cluster 0* obtained from K-Means.

Pathway Name	*p* Value	FDR Correction	Bonferroni Correction
(1) Metabolism of proteins	$4.48\times 10^{-216}$	$8.00\times 10^{-213}$	$8.00\times 10^{-213}$
(2) Signaling pathways	$4.05\times 10^{-204}$	$3.62\times 10^{-201}$	$7.23\times 10^{-201}$
(3) Gene expression (transcription)	$8.23\times 10^{-172}$	$4.90\times 10^{-169}$	$1.47\times 10^{-168}$
(4) RNA polymerase II transcription	$5.12\times 10^{-133}$	$2.29\times 10^{-130}$	$9.15\times 10^{-130}$
(5) Immune system	$1.35\times 10^{-125}$	$4.81\times 10^{-123}$	$2.41\times 10^{-122}$
(6) Metabolism	$3.93\times 10^{-122}$	$1.17\times 10^{-119}$	$7.03\times 10^{-119}$
(7) Post-translational protein modification	$1.73\times 10^{-121}$	$4.41\times 10^{-119}$	$3.09\times 10^{-118}$
(8) Cellular responses to stimuli	$5.96\times 10^{-109}$	$1.33\times 10^{-106}$	$1.07\times 10^{-105}$
(9) Cellular responses to stress	$3.57\times 10^{-107}$	$7.09\times 10^{-105}$	$6.38\times 10^{-104}$
(10) Developmental Biology	$3.10\times 10^{-82}$	$5.04\times 10^{-80}$	$5.55\times 10^{-79}$

## Data Availability

The dataset, the Python code to reproduce to results of this work and the example results are available at https://github.com/mmilano87/PCAPxDEG (accessed on 3 October 2022).

## Abbreviations

The following abbreviations are used in this manuscript:

DEG	Differential Expresses Gene
SNP	Single Polymorphism Nucleotide
mRNA	messenger RNA
DBSCAN	Density-Based Spatial Clustering of Applications with Noise
PCA	Principal Component Analysis
SSE	Sum of the Squared Error
BiP	BioPax Parser
cPEA	cross Pathway Enrichment Analysis
SVD	Singular Value Decomposition

## Appendix A

In this appendix, we will describe the meaning of the Python statements used to perform clustering analysis on gene expression data sets. We assume that the users have already installed Python and all the necessary additional packages on their machines. For more information on setting up a Python environment for machine learning in Windows, Linux and macOS, the readers could go through the Python user guides related to their installed operating system.

The first lines of code of any Python script identify the import section, and it is mandatory to include in the script the necessary additional library, i.e., the sklearn, numpy, matplolib, seaborn and pandas supplied as additional functions to the programmer. In Listings A1 and A2, the import section starts at line 1 and ends at line 8. In this way, all the classes and functions of each module can be used by a simple function call; e.g., to show a plot on the screen by using the matplot functions, the following statement can be used: plt.show(). It is worthwhile to note that each library other than the one built in must be explicitly imported into the script before they can be used.

**Listing A1.** Python script to analyze gene expression data sets using the K-Means algorithm available in the *scikit-learn* package.

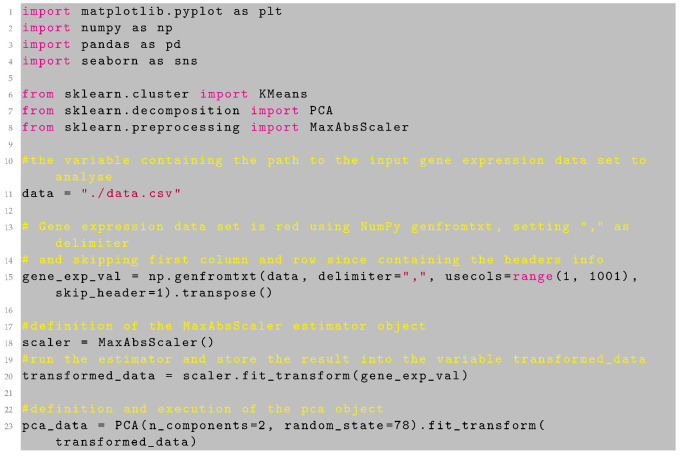

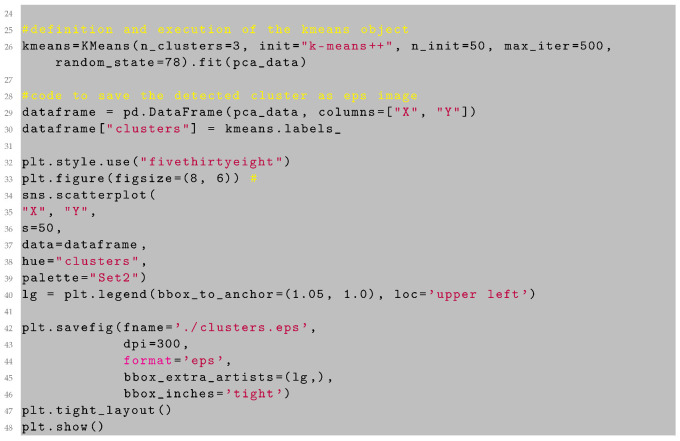

**Listing A2.** Python script to analyze gene expression data sets using the DBSCAN algorithm available in the *scikit-learn* package.

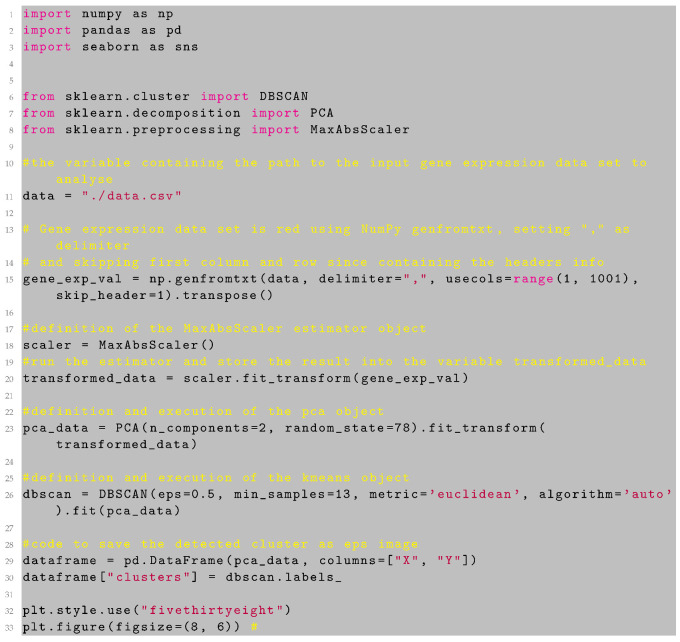

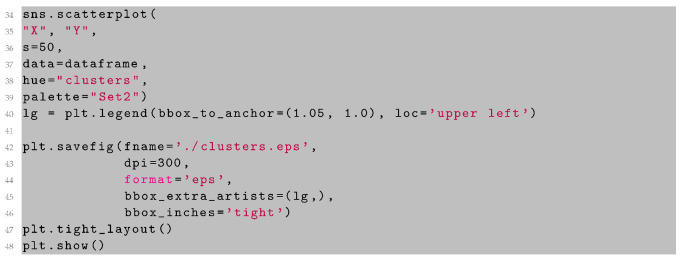

The instruction at line 11 of both Listings A1 and A2 defines the variable named data that holds the path to the input data to investigate. To upload the input data, the data variable is passed as argument to the genfromtxt*numpy* function that will load, transpose and memorize the input data within the variable named *gene_exp_val*. The other arguments of the genfromtxt are: *(i) delimiter=* that defines symbols used to delimit the input data, e.g., the *’,’*; *(ii) usecols=* allows selecting the range of columns to upload, e.g., all the columns; *(iii) skip_header=* we pass the index of the headers row, which is not useful for the clustering analysis. At the end of data uploading, using the numpy transpose functions, the input table will be transposed and stored into the *gene_exp_val* variable in Listings A1 and A2 line 15.

Data scaling is obtained by instantiating a MaxAbsScaler object using the statement at Listings A1 and A2 line 18 called scaler. Next, we invoke on the scaler object the fit_transform(gene_exp_val) and pass the data within the gene_exp_val variable. As a result, the scaled data will be stored into the transformed_data variable Listings A1 and A2 line 20. Dimensionality reduction is obtained creating an object of the class PCA calling the constructor. The constructor allows setting up the value of the parameters n_componets = 2, e.g., the number of components to keep and random_state = 78, e.g., an int value allows reproducing results across multiple function calls. Next, we use the fit_transform(transformed_data) method, feeding it with the transformed data within the transformed_data variable Listings A1 and A2 line 23.

The preprocessed DEG data are held within the variable pca_data that will be supplied as input data to both the *K-Means* object Listing A1 line 26 and the *DBSCAN* objects Listing A2 line 26. Now, all the information about the *K-Means* execution is memorized within the variable named kmeans; in particular, we are interested in the labels_ attribute value. In addition, for DBSCAN, the information is stored within the variable named dbscan; in particular, we are interested in the labels_ attribute value.

The values of labels_ of both objects will be used to create and fill the columns of a data frame line 30 of both Listings A1 and A2 to generate the cluster figure through the *matplot* and *seaborn* library lines 32…48. The statement at line 32 allows choosing the figure style, whereas line 33 allows tuning the figure size. The statement at line 34 spread on multiple lines just for indentation purposes defines the type of *seaborn* plot to create, e.g., *scatterplot* calling the scatterplot function and customizing its parameters as follows. The “X” and “Y” values define the name of the *x* and *y* axes in the plot. The argument s=50 defines the size of scatter markers. The argument data=dataframe allows tuning the input data structure, whereas hue="clusters" produces points with different colors, and the palette="Set2" parameter defines the colors to use when mapping the hue parameters.

The statement at line 42 of both Listings A1 and A2 allows saving the figure as a file on the hard disk. The savefig function allows saving the figure in a different format by setting the format argument; e.g., format=’eps’ saves the figure on the disk as an eps file at the location defined by the path held by the fname argument, e.g., ’./clusters.eps’. The dpi=300 argument defines the resolution in dots per inch. The bbox_extra_artists argument allows defining the extra element to include into the figure list of the Artist, which is optional when the tight bbox is calculated, e.g., the legend lg defined at line 40. The bbox_inches=’tight’ defines the portion of the figure to save. The statement at line 47 allows adjusting the padding between and around subplots, and finally, the statement at line 48 visualizes the screen the scatter plot.

Finally, it is worth noting how simple it is to perform clustering analysis using different algorithms in Python. The main steps of both data preprocessing and post-processing are the same if using the K-Means or DBSCAN method; the only difference is the use of K-Means or DBSCAN at line 26 of both Listings A1 and A2. In this way, it is straightforward to analyze DEG data sets through several different clustering algorithms, allowing researchers to chose the algorithm that provides the best results in terms of predicted clusters and the quality of clusters, contributing to detecting genes groups affecting the underlying cellular functions to provide new actionable knowledge to provide new treatments as well as to discover new bio-markers.

## References

[1] Arbitrio M., Di Martino M.T., Scionti F., Agapito G., Guzzi P.H., Cannataro M., Tassone P., Tagliaferri P. (2016). DMET™(Drug Metabolism Enzymes and Transporters): A pharmacogenomic platform for precision medicine. Oncotarget.

[2] Arbitrio M., Scionti F., Altomare E., Di Martino M.T., Agapito G., Galeano T., Staropoli N., Iuliano E., Grillone F., Fabiani F. (2019). Polymorphic Variants in NR 1I3 and UGT 2B7 Predict Taxane Neurotoxicity and Have Prognostic Relevance in Patients With Breast Cancer: A Case-Control Study. Clin. Pharmacol. Ther..

[3] Heller M.J. (2002). DNA microarray technology: Devices, systems, and applications. Annu. Rev. Biomed. Eng..

[4] Arbitrio M., Di Martino M.T., Barbieri V., Agapito G., Guzzi P.H., Botta C., Iuliano E., Scionti F., Altomare E., Codispoti S. (2016). Identification of polymorphic variants associated with erlotinib-related skin toxicity in advanced non-small cell lung cancer patients by DMET microarray analysis. Cancer Chemother. Pharmacol..

[5] Di Martino M.T., Scionti F., Sestito S., Nicoletti A., Arbitrio M., Guzzi P.H., Talarico V., Altomare F., Sanseviero M.T., Agapito G. (2016). Genetic variants associated with gastrointestinal symptoms in Fabry disease. Oncotarget.

[6] Carter N.P. (2007). Methods and strategies for analyzing copy number variation using DNA microarrays. Nat. Genet..

[7] Bier F.F., Nickisch-Rosenegk M.v., Ehrentreich-Foerster E., Reiss E., Henkel J., Strehlow R., Andresen D. (2007). DNA microarrays. Biosensing for the 21st Century.

[8] Mills K. (2005). Analysis of microarray data. Oxidative Stress Dis..

[9] Guzzi P.H., Agapito G., Milano M., Cannataro M. (2016). Methodologies and experimental platforms for generating and analysing microarray and mass spectrometry-based omics data to support P4 medicine. Briefings Bioinform..

[10] Peterson L.E. (2013). Classification analysis of DNA Microarrays.

[11] Piatetsky-Shapiro G., Tamayo P. (2003). Microarray data mining: Facing the challenges. ACM SIGKDD Explor. Newsl..

[12] Verducci J.S., Melfi V.F., Lin S., Wang Z., Roy S., Sen C.K. (2006). Microarray analysis of gene expression: Considerations in data mining and statistical treatment. Physiol. Genom..

[13] Salem D.A., Seoud R., Ali H.A. Dmca: A combined data mining technique for improving the microarray data classification accuracy. Proceedings of the 2011 International Conference on Environment and Bioscience.

[14] Baldi P., Hatfield G.W. (2011). DNA Microarrays and Gene expression: From Experiments to Data Analysis and Modeling.

[15] Agapito G., Pastrello C., Guzzi P.H., Jurisica I., Cannataro M. (2020). BioPAX-Parser: Parsing and enrichment analysis of BioPAX pathways. Bioinformatics.

[16] Agapito G., Cannataro M. (2020). cPEA: A parallel method to perform pathway enrichment analysis using multiple pathways databases. Soft Comput..

[17] Agapito G., Milano M., Cannataro M. (2021). Parallel Network Analysis and Communities Detection (PANC) Pipeline for the Analysis and Visualization of COVID-19 Data. Parallel Process. Lett..

[18] Miao Z., Qi Y., Qian A., Yang T. (2016). Data Mining of Differentially Expressed Genes Based on Gene Expression Profiling Microarray. Rev. Téc. Ing. Univ. Zulia..

[19] Keller A., Leidinger P., Borries A., Wendschlag A., Wucherpfennig F., Scheffler M., Huwer H., Lenhof H.P., Meese E. (2009). miRNAs in lung cancer-studying complex fingerprints in patient’s blood cells by microarray experiments. BMC Cancer.

[20] Nancy J.Y., Khanna N.H., Kannan A. (2017). A bio-statistical mining approach for classifying multivariate clinical time series data observed at irregular intervals. Expert Syst. Appl..

[21] Terkelsen T., Krogh A., Papaleo E. (2020). CAncer bioMarker Prediction Pipeline (CAMPP)—A standardized framework for the analysis of quantitative biological data. PLoS Comput. Biol..

[22] Pastrello C., Otasek D., Fortney K., Agapito G., Cannataro M., Shirdel E., Jurisica I. (2013). Visual data mining of biological networks: One size does not fit all. PLoS Comput. Biol..

[23] Agapito G., Guzzi P.H., Cannataro M. (2015). DMET-Miner: Efficient discovery of association rules from pharmacogenomic data. J. Biomed. Inform..

[24] Kuo W.P., Kim E.Y., Trimarchi J., Jenssen T.K., Vinterbo S.A., Ohno-Machado L. (2004). A primer on gene expression and microarrays for machine learning researchers. J. Biomed. Inform..

[25] Zhang L., Mao R., Lau C.T., Chung W.C., Chan J.C., Liang F., Zhao C., Zhang X., Bian Z. (2022). Identification of useful genes from multiple microarrays for ulcerative colitis diagnosis based on machine learning methods. Sci. Rep..

[26] Cho S.B., Won H.H. Machine learning in DNA microarray analysis for cancer classification. Proceedings of the First Asia-Pacific Bioinformatics Conference on Bioinformatics 2003.

[27] Tabares-Soto R., Orozco-Arias S., Romero-Cano V., Bucheli V.S., Rodríguez-Sotelo J.L., Jiménez-Varón C.F. (2020). A comparative study of machine learning and deep learning algorithms to classify cancer types based on microarray gene expression data. PeerJ Comput. Sci..

[28] Wang Y., Tetko I.V., Hall M.A., Frank E., Facius A., Mayer K.F., Mewes H.W. (2005). Gene selection from microarray data for cancer classification—a machine learning approach. Comput. Biol. Chem..

[29] Guzzi P.H., Agapito G., Di Martino M.T., Arbitrio M., Tassone P., Tagliaferri P., Cannataro M. (2012). DMET-analyzer: Automatic analysis of Affymetrix DMET data. BMC Bioinform..

[30] Cui X., Churchill G.A. (2003). Statistical tests for differential expression in cDNA microarray experiments. Genome Biol..

[31] Simon R.M., Korn E.L., McShane L.M., Radmacher M.D., Wright G.W., Zhao Y. (2003). Design and Analysis of DNA Microarray Investigations.

[32] Owzar K., Barry W.T., Jung S.H., Sohn I., George S.L. (2008). Statistical challenges in preprocessing in microarray experiments in cancer. Clin. Cancer Res..

[33] Barlow H.B. (1989). Unsupervised learning. Neural Comput..

[34] Rueda L., Qin L. An unsupervised learning scheme for dna microarray image spot detection. Proceedings of the First International Conference on Complex Medical Engineering.

[35] Boutros P.C., Okey A.B. (2005). Unsupervised pattern recognition: An introduction to the whys and wherefores of clustering microarray data. Briefings Bioinform..

[36] Saha I., Maulik U., Bandyopadhyay S., Plewczynski D. (2011). Unsupervised and supervised learning approaches together for microarray analysis. Fundam. Inform..

[37] Fratello M., Cattelani L., Federico A., Pavel A., Scala G., Serra A., Greco D. (2022). Unsupervised Algorithms for Microarray Sample Stratification. Microarray Data Analysis.

[38] Shannon W., Culverhouse R., Duncan J. (2003). Analyzing microarray data using cluster analysis. Pharmacogenomics.

[39] Das A.K., Pati S.K., Chakrabarty S. Reduct generation of microarray dataset using rough set and graph theory for unsupervised learning. Proceedings of the Second International Conference on Computational Science, Engineering and Information Technology.

[40] Ma P.C., Chan K.C., Yao X., Chiu D.K. (2006). An evolutionary clustering algorithm for gene expression microarray data analysis. IEEE Trans. Evol. Comput..

[41] Kim D., Cho K.H. (2022). Hidden patterns of gene expression provide prognostic insight for colorectal cancer. Cancer Gene Ther..

[42] Kellgren T. (2020). Hidden Patterns That Matter: Statistical Methods for Analysis of DNA and RNA Data. Ph.D. Thesis.

[43] Quackenbush J. (2001). Computational analysis of microarray data. Nat. Rev. Genet..

[44] Yin R., Zhou X., Zheng J., Kwoh C.K. (2018). Computational identification of physicochemical signatures for host tropism of influenza A virus. J. Bioinform. Comput. Biol..

[45] Zeng M., Zhang F., Wu F.X., Li Y., Wang J., Li M. (2020). Protein–protein interaction site prediction through combining local and global features with deep neural networks. Bioinformatics.

[46] Kwan H.K., Arniker S.B. Numerical representation of DNA sequences. Proceedings of the 2009 IEEE International Conference on Electro/Information Technology.

[47] Adetiba E., Olugbara O.O., Taiwo T.B. (2016). Identification of pathogenic viruses using genomic cepstral coefficients with radial basis function neural network. Advances in Nature and Biologically Inspired Computing.

[48] Rui Y., Luo Z., Kwoh C.K. Alignment-free machine learning approaches for the lethality prediction of potential novel human-adapted coronavirus using genomic nucleotide. bioRxiv.

[49] Hackstadt A.J., Hess A.M. (2009). Filtering for increased power for microarray data analysis. BMC Bioinform..

[50] Quackenbush J. (2002). Microarray data normalization and transformation. Nat. Genet..

[51] Liberti L., Lavor C., Maculan N., Mucherino A. (2014). Euclidean distance geometry and applications. SIAM Rev..

[52] Craw S., Sammut C., Webb G.I. (2017). Manhattan Distance. Encyclopedia of Machine Learning and Data Mining.

[53] Cantrell C.D. (2000). Modern Mathematical Methods for Physicists and Engineers.

[54] Lahitani A.R., Permanasari A.E., Setiawan N.A. Cosine similarity to determine similarity measure: Study case in online essay assessment. Proceedings of the 2016 4th International Conference on Cyber and IT Service Management.

[55] Ivchenko G., Honov S. (1998). On the jaccard similarity test. J. Math. Sci..

[56] Annathurai K.S., Angamuthu T. (2022). Sorensen-dice similarity indexing based weighted iterative clustering for big data analytics. Int. Arab J. Inf. Technol..

[57] Lloyd S.P. (1982). Least squares quantization in PCM. IEEE Trans. Inf. Theory.

[58] Ester M., Kriegel H.P., Sander J., Xu X. (1996). A Density-Based Algorithm for Discovering Clusters in Large Spatial Databases with Noise. Proceedings of the Second International Conference on Knowledge Discovery and Data Mining.

[59] Barrett T., Troup D.B., Wilhite S.E., Ledoux P., Rudnev D., Evangelista C., Kim I.F., Soboleva A., Tomashevsky M., Edgar R. (2007). NCBI GEO: Mining tens of millions of expression profiles—database and tools update. Nucleic Acids Res..

[60] Barrett T., Suzek T.O., Troup D.B., Wilhite S.E., Ngau W.C., Ledoux P., Rudnev D., Lash A.E., Fujibuchi W., Edgar R. (2005). NCBI GEO: Mining millions of expression profiles—database and tools. Nucleic Acids Res..

[61] Scionti F., Agapito G., Caracciolo D., Riillo C., Grillone K., Cannataro M., Di Martino M.T., Tagliaferri P., Tassone P., Arbitrio M. (2022). Risk Alleles for Multiple Myeloma Susceptibility in ADME Genes. Cells.

[62] Rahmati S., Abovsky M., Pastrello C., Kotlyar M., Lu R., Cumbaa C.A., Rahman P., Chandran V., Jurisica I. (2020). pathDIP 4: An extended pathway annotations and enrichment analysis resource for human, model organisms and domesticated species. Nucleic Acids Res..

[63] Kulkoyluoglu-Cotul E., Arca A., Madak-Erdogan Z. (2019). Crosstalk between Estrogen Signaling and Breast Cancer Metabolism. Trends Endocrinol. Metab..

[64] Zhang D., Wang G., Wang Y. (2014). Transcriptional regulation prediction of antiestrogen resistance in breast cancer based on RNA polymerase II binding data. BMC Bioinform..

[65] Harold C.M., Buhagiar A.F., Cheng Y., Baserga S.J. (2021). Ribosomal RNA transcription regulation in breast cancer. Genes.

[66] Liu J., Du J., Li Y., Wang F., Song D., Lin J., Li B., Li L. (2022). Catalpol induces apoptosis in breast cancer in vitro and in vivo: Involvement of mitochondria apoptosis pathway and post-translational modifications. Toxicol. Appl. Pharmacol..

[67] Yu G., Jiang L., Xu Y., Guo H., Liu H., Zhang Y., Yang H., Yuan C., Ma J. (2012). Silencing prion protein in MDA-MB-435 breast cancer cells leads to pleiotropic cellular responses to cytotoxic stimuli. PLoS ONE.

[68] Hannun Y.A. (1996). Functions of ceramide in coordinating cellular responses to stress. Science.

[69] Jiang X., Shapiro D.J. (2014). The immune system and inflammation in breast cancer. Mol. Cell. Endocrinol..

[70] Furth P.A., Nakles R.E., Millman S., Diaz-Cruz E.S., Cabrera M.C. (2011). Signal transducer and activator of transcription 5 as a key signaling pathway in normal mammary gland developmental biology and breast cancer. Breast Cancer Res..

